# Chromosomal microarray and whole‐exome sequence analysis in Taiwanese patients with autism spectrum disorder

**DOI:** 10.1002/mgg3.996

**Published:** 2019-10-08

**Authors:** Ya‐Sian Chang, Chien‐Yu Lin, Hsi‐Yuan Huang, Jan‐Gowth Chang, Haung‐Tsung Kuo

**Affiliations:** ^1^ Epigenome Research Center China Medical University Hospital Taichung Taiwan; ^2^ Department of Laboratory Medicine China Medical University Hospital Taichung Taiwan; ^3^ Center for Precision Medicine China Medical University Hospital Taichung Taiwan; ^4^ Department of Medical Laboratory Science and Biotechnology China Medical University Taichung Taiwan; ^5^ Graduate Institute of Clinical Medical Science and School of Medicine China Medical University Taichung Taiwan; ^6^ Departments of Laboratory Medicine Taichung Tzu Chi Hospital Buddhist Tzu Chi Medical Foundation Taichung Taiwan; ^7^ Graduate Institute of Biomedical Sciences School of Medicine China Medical University Taichung Taiwan; ^8^ Department of Bioinformatics and Medical Engineering Asia University Taichung Taiwan; ^9^ Department of Developmental and Behavioral Pediatrics Children's Hospital of China Medical University Taichung Taiwan

**Keywords:** Autism spectrum disorder, Chromosomal microarray analysis, Copy number variant, Whole‐exome sequencing

## Abstract

**Background:**

Autism spectrum disorder (ASD) is defined as a group of genetically and clinically heterogeneous neurodevelopmental disorders. Interplay between de novo and inherited rare variants has been suspected in the development of ASD.

**Methods:**

Here, we applied 750K oligonucleotide microarray analysis and whole‐exome sequencing (WES) to five trios from Taiwanese families with ASD.

**Results:**

The chromosomal microarray analysis revealed three representative known diagnostic copy number variants that contributed to the clinical presentation: the chromosome locations 2q13, 1q21.1q21.2, and 9q33.1. WES detected 22 rare variants in all trios, including four that were newly discovered, one of which is a de novo variant. Sequencing variants of *JMJD1C*, *TCF12*, *BIRC6*, and *NHS* have not been previously reported. A novel de novo variant was identified in *NHS* (p.I7T). Additionally, seven pathogenic variants, including *SMPD1*, *FUT2*, *BCHE*, *MYBPC3*, *DUOX2*, *EYS*, and *FLG*, were detected in four probands. One of the involved genes, *SMPD1*, had previously been reported to be mutated in patients with Parkinson's disease.

**Conclusions:**

These findings suggest that de novo or inherited rare variants and copy number variants may be double or multiple hits of the probands that lead to ASD. WES could be useful in identifying possible causative ASD variants.

## Graphical abstract

We applied 750K oligonucleotide microarray analysis and whole‐exome sequencing to five trios from Taiwanese families with ASD. A novel de novo variant was identified in NHS (c.20T>C; p.I7T). de novo or inherited rare variants and copy number variants may be double or multiple hits of the probands that lead to ASD.

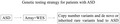

## 1 INTRODUCTION

Autism spectrum disorder (ASD), which belongs to a group of neurobehavioral syndromes, is characterized by significantly impaired social interaction and communication as well as by restricted, repetitive, and stereotyped patterns of behaviors, interests, and activities (Johnson, Myers, & American Academy of Pediatrics Council on Children With, 2007). The prevalence of ASD is estimated to be 1:59 children and 1:100 adults (Baio et al., 2018; Brugha et al., 2011). The rate of ASD is higher in males than in females (4:1), which is higher than those of Down syndrome and epilepsy. Developmental delays are observed in approximately 40% of individuals with ASD, and approximately 70% show some level of intellectual disability. ASD has strong genetic contributions, and single‐gene disorders are recognized as causative in less than 20% of ASD cases (Herman et al., 2007). The most consistently reported single gene disorders associated with ASD are fragile X syndrome, Rett syndrome, and tuberous sclerosis. The prevalence of fragile X syndrome among subjects with ASD is 1.5%–3% (Clifford et al., 2007). The genetic etiology of ASD is complex.

Chromosomal microarray analysis (CMA) examines gross chromosomal structural abnormalities and can detect deletions and duplications as well as the size and presence of known genes within a chromosomal region. The most common microarray abnormalities in ASD involve the chromosome regions 15q11‐q13, 16p11.2, and 22q11.2 (Carter & Scherer, 2013; Roberts, Hovanes, Dasouki, Manzardo, & Butler, 2014). In the clinical setting, CMA, which has a diagnostic yield ranging from 7.0% to 9.0%, is recommended as the first tier test for children and adults presenting with ASD (Battaglia et al., 2013; McGrew, Peters, Crittendon, & Veenstra‐Vanderweele, 2012; Shen et al., 2010).

Technological improvements have led to tremendous advances in our understanding of the genetic basis of ASD over the past 10 years. Most genomic studies on ASD using next‐generation sequencing (NGS) have focused on protein‐coding regions and analyzed trio information to identify sequence‐level de novo mutations (De Rubeis et al., 2014; Iossifov et al., 2014, 2012; Neale et al., 2012; O'Roak et al., 2012; Sanders et al., 2012). Hundreds of genes have been implicated in the cause of ASD. The identification of new genes involved in ASD has made this condition a strong candidate for genome‐based diagnostic testing, which consists of CMA and NGS, as well as whole‐genome sequencing (WGS) and whole‐exome sequencing (WES).

Recently, Guo et al. applied WGS, WES, and CMA to investigate genomic variants in ASD families and compared the performances of WGS and WES for use in diagnostic testing (Guo et al., 2019). The authors reported the diagnostic utility of WGS for detecting disorder‐related variants (particularly multiple rare‐risk variants that contribute to phenotypic severity in individuals with ASD), identifying genetic heterogeneity in multiplex ASD families and predicting novel ASD‐associated genes for future study.

In this study, we aimed to define causative or susceptibility variants for ASD and their copy number variants by CMA. We studied five subjects who are typical of those seen in developmental pediatric clinics. The sample was stratified based on the clinical phenotype of the patients.

## 2 MATERIALS AND METHODS

### 2.1 Subjects with ASD

Five patients with a clinical diagnosis of ASD were enrolled in the study. Autism screening was performed using the Autism Behavior Checklist, Taiwanese version (ABC‐T), which was modified from the third edition of the Autism Behavior Checklist of Autism Screening Instrument for Education Planning (Krug, Arick, & Almond, 1980). Family members were also enrolled for inheritance pattern analysis. Blood samples were obtained, and genomic DNA was extracted using the Nucleospin® Blood Kit (Macherey‐Nagel, GmbH & Co. KG, Duren, Germany). This study was approved by the China Medical University Hospital (CMUH105‐REC1‐039).

### 2.2 Single‐nucleotide polymorphism (SNP) array analysis

DNA samples (250 ng) were hybridized to the Affymetrix CytoScan 750K array according to the manufacturer's instructions. The 750K array contained greater than 750,000 markers for copy number analysis and 200,000 SNP probes for genotyping. The following standard experimental procedures were performed: digestion, ligation, polymerase chain reaction (PCR), PCR purification, fragmentation, labeling, hybridization, washing, staining, and scanning. After hybridization, GeneChip Scanner 3000 7G, Affymetrix GeneChip Command Console software, and Affymetrix ChAS 2.0 software were used for scanning the arrays, extracting the images, and performing the analysis, respectively. All data had to pass quality control (QC) metrics including the median of the absolute values of all pairwise differences ≤ 0.30, SNPQC ≥ 15, and a waviness standard deviation ≤ 0.12.

### 2.3 WES

In total, 100 ng of genomic DNA based on Qubit quantification was mechanically fragmented on a M220 focused ultrasonicator Covaris (Covaris, Woburn, MA, USA), and QC was performed using an Agilent Bioanalyzer 4200 (Agilent Technologies, Santa Clara, CA, USA) to ensure an average fragment size of 150–200 bp. End repair, A‐tailing, adaptor ligation, and enrichment of DNA fragments were then performed. A 200–400 bp band was gel‐selected, and exome capture was performed using a TruSeq Exome Library Preparation Kit (Illumina, San Diego, CA, USA). The DNA library was quantified in the Qubit 3.0 Fluorometer (Invitrogen) and Agilent 4200 Bioanalyzer (Agilent Technologies). Samples were sequenced on an Illumina NextSeq500 platform and 150‐bp paired‐end reads were generated.

### 2.4 Data analysis

Base calling and quality scoring were performed by an updated implementation of real‐time analysis on the NextSeq500 system. Bcl2fastq Conversion Software was used to demultiplex data and convert the BCL files to FASTQ files. Sequenced reads were trimmed for low‐quality sequences and mapped to the human reference genome (hg19) using the Burrows–Wheeler alignment (Li & Durbin, 2009). Finally, SNPs and small insertions/deletions were detected using Genome Analysis Toolkit and VarScan using their default settings (Koboldt et al., 2012; McKenna et al., 2010). ANNOVAR was used to annotate the VCF files by gene, region, and filters from several other databases (Wang, Li, & Hakonarson, 2010). Finally, we annotated the mutations using several databases and tools, including dbSNP (build 147), GnomAD (http://gnomad-old.broadinstitute.org/), Denovo‐db (http://denovo-db.gs.washington.edu/denovo-db/), ClinVar, Polyphen‐2, SIFT, and CADD (Adzhubei et al., 2010; Kircher et al., 2014; Kumar, Henikoff, & Ng, 2009; Landrum et al., 2014; Sherry et al., 2001; Turner et al., 2017). Pathways were analyzed using STRING (https://string-db.org). Additionally, ASD‐related genes reported in the public databases OMIM and AutDB were selected (Basu, Kollu, & Banerjee‐Basu, 2009).

### 2.5 Variant validations and segregation analysis

We used PCR and Sanger sequencing to validate candidate variants from WES. Segregation analysis was carried out on family members. PCR primers were designed using Primer3 (http://bioinfo.ut.ee/primer3-0.4.0/). Table S1 lists the designed primers. The products were directly sequenced with an ABI PRISM BigDye kit using an ABI 3130 DNA sequencer (Applied Biosystems). Sequencing results were analyzed using the software Chromas, version 2.23.

## 3 RESULTS

Following QC of the WES data, five probands were analyzed further and confirmed by Sanger sequencing. For these, a mean coverage depth of 141X was achieved (Table 1). Patient 1, a 21‐year‐old male who presented with autism combined with epilepsy, had an ABC‐T score of 28 (Table 3). The 750K microarray showed a 2q13 duplication (482.154 kbp) containing three OMIM genes (*RGPD6*, *MALL*, and *NPHP1*). WES revealed four rare variants in *SHANK3* (c.3658A > G; p.T1220A; rs751183635), *DNAH10* (c.2800C > T; p.R934C; rs757691040), *ESR2* (c.1228C > T; p.R410C; rs528840784), and *NAALADL2* (c.1424G > A; p.R475H; rs372908344) (Table 2). Among them, SHANK3 and ESR2 are involved in negative regulation of signal transduction (GO:0,009,968). Furthermore, three pathogenic mutations were observed, *SMPD1* (p.P186L), *FUT2* (p.R202X), and *BCHE* (p.T343fs).

**Table 1:** Whole‐exome sequencing alignment and mean base depth statistics for 5 probands for the analysis

Case	Total raw reads	Total effective reads	Reads mapped to genome	Average read depth of target regions	Number of SNVs on target
ASD23	172,869,310	159,621,404	159,588,187	139.246	34,233
ASD24	186,622,966	172,125,108	172,094,954	143.877	34,284
ASD25	192,186,388	173,661,734	173,616,740	126.054	34,093
ASD26	191,244,742	176,442,474	176,407,661	154.055	34,942
ASD27	167,228,010	153,870,614	153,839,987	141.935	33,945
Average	182,030,283	167,144,267	167,109,506	141.033	34,299

**Table 2:** Summary of the rare variants in autism‐related genes detected in this study

Proband ID (Gender)	Mode of Inheritance	Identified Variant			Genotype			Functional prediction			MAF	Abnormal chromosomal microarray data				
		Gene	Base Change	Amino Acid Change	Proband	UF	UM	PolyPhen2	SIFT	CADD	ExAC/1000 Genome project/GnomAD/Denovo‐db	Del/Dup	Chromosome location	Chromosome coordinates		Number of genes in deletion or duplication
ASD23 (M)	AD	*SHANK3*	c.3658A > G	p.T1220A	A/G	A/G	A/A	NA	NA	NA	0	Dup	2q13	110,498,141–110,980,251		3
	AD	*DNAH10*	c.2800C > T	p.R934C	C/T	C/T	C/C	Probably damaging	Deleterious	34	0.0001					
	AD	*ESR2*	c.1228C > T	p.R410C	C/T	C/C	C/T	Probably damaging	Deleterious	23.3	0.0006					
	AD	*NAALADL2*	c.1424G > A	p.R475H	G/A	G/A	G/G	Possibly damaging	Deleterious	23.1	0.0008					
ASD24 (M)	AD	*DLGAP3*	c.1759G > C	p.G587R	G/C	G/C	G/G	Possibly damaging	Deleterious	27	0.0004	Not detected				
	AD	*SLC1A2*	c.1091G > A	p.R364H	G/A	G/A	G/G	Probably damaging	Deleterious	35	0.0001					
	AD	*CLTCL1*	c.1061G > A	p.R354H	G/A	G/A	G/G	Probably damaging	Deleterious	24.9	0.0009					
ASD25 (M)	AD	*WFS1*	c.2144G > T	p.S715I	G/T	G/G	G/T	Possibly damaging	Deleterious	25	0.0002	Dup	1q21.1q21.2	145,895,746–147,844,777		10
	AD	*TNN*	c.1681T > C	p.Y561H	T/C	T/T	T/C	Probably damaging	Deleterious	25.7	0.0001					
	AD	*JMJD1C*	ac.6344A > C	p.F2115C	A/C	A/C	A/A	Probably damaging	Deleterious	29.9						
	AD	*APP*	c.1748A > G	p.E583G	A/G	A/A	A/G	Possibly damaging	Deleterious	28.8	0.0001					
	AD	*SYNE1*	c.9878C > T	p.S3293F	C/T	C/T	C/C	Probably damaging	Deleterious	32	0.0005					
	AD	*MPP6*	c.61G > A	p.D21N	G/A	G/G	G/A	Probably damaging	Deleterious	33	0.0003					
	AD	*MCC*	c.60_61insAGC	p.G21delinsSG	Het	WT	Het	NA	NA	NA	0					
ASD26 (M)	AD	*TSC2*	c.5418T > G	p.F1806L	T/G	T/G	T/T	Probably damaging	Deleterious	23.2	0.0001	Not detected				
	AD	*SETBP1*	c.2842C > T	p.R948C	C/T	C/T	C/C	Probably damaging	Deleterious	24.5	0.0002					
	AD	*TCF12*	ac.770C > T	p.R257H	C/T	C/C	C/T	Probably damaging	Deleterious	28.1						
	AD	*LZTS2*	c.1259G > A	p.R420Q	G/A	G/A	G/G	Probably damaging	Deleterious	28.6	0.0003					
	AD	*BIRC6*	ac.6600G > T	p.Q2200H	G/T	G/T	G/G	Probably damaging	Deleterious	26.7						
	AD	*EPHA6*	c.527A > C	p.N176T	A/C	A/C	A/A	Probably damaging	Deleterious	24.9	0.00003					
	X‐Y pseudoautosomal	*ASMT*	c.451G > A	p.G151S	G/A	G/A	G/G	NA	NA	NA	0.0005					
ASD27 (M)	De novo	*NHS*	ac.20T > C	p.I7T	C/C	T/T	T/C	Probably damaging	Deleterious	23.9		Dup	9q33.1	118,921,750–120,012,115	3	

Abbreviations: UF, unaffected father; UM, unaffected mother.

a Variants were not reported.

Patient 2, an 8‐year‐old male, had an ABC‐T score of 27 (Table 3). The 750K microarray revealed no abnormalities. WES revealed three rare variants in *DLGAP3* (c.1759G > C; p.G587R; rs762072609), *SLC1A2* (c.1091G > A; p.R364H; rs147645566), and *CLTCL1* (c.1061G > A; p.R354H; rs201506683) (Table 2). Additionally, two pathogenic mutations were observed, *MYBPC3* (p.E334K) and *DUOX2* (p.K530X).

**Table 3:** Summary of the phenotypic features of patients with autism spectrum disorder and the relevant findings of the study

Patient	Rare variants in autism‐related genes involved pathways^a^				ABCT (47 items)					
	Negative regulation of Wnt signaling pathway (GO:0030178)	Negative regulation of canonical Wnt signaling pathway (GO:0090090)	Negative regulation of signal transduction (GO:0009968)	Endoplasmic reticulum calcium ion homeostasis (GO:0032469)	Sensory (8 items)	Relating (11 items)	Body and object use (12 items)	Language (8 items)	Social and self‐help (8 items)	Total score
ASD23			+		3	6	9	4	6	28
ASD24					1	6	7	6	7	27
ASD25	+	+	+	+	3	4	3	5	5	20
ASD26	+	+	+		4	4	8	5	6	27
ASD27					4	8	12	6	6	36

a Rare variants in autism‐related genes (as shown in Table 2) are involved in several pathways.

**Figure 1:**
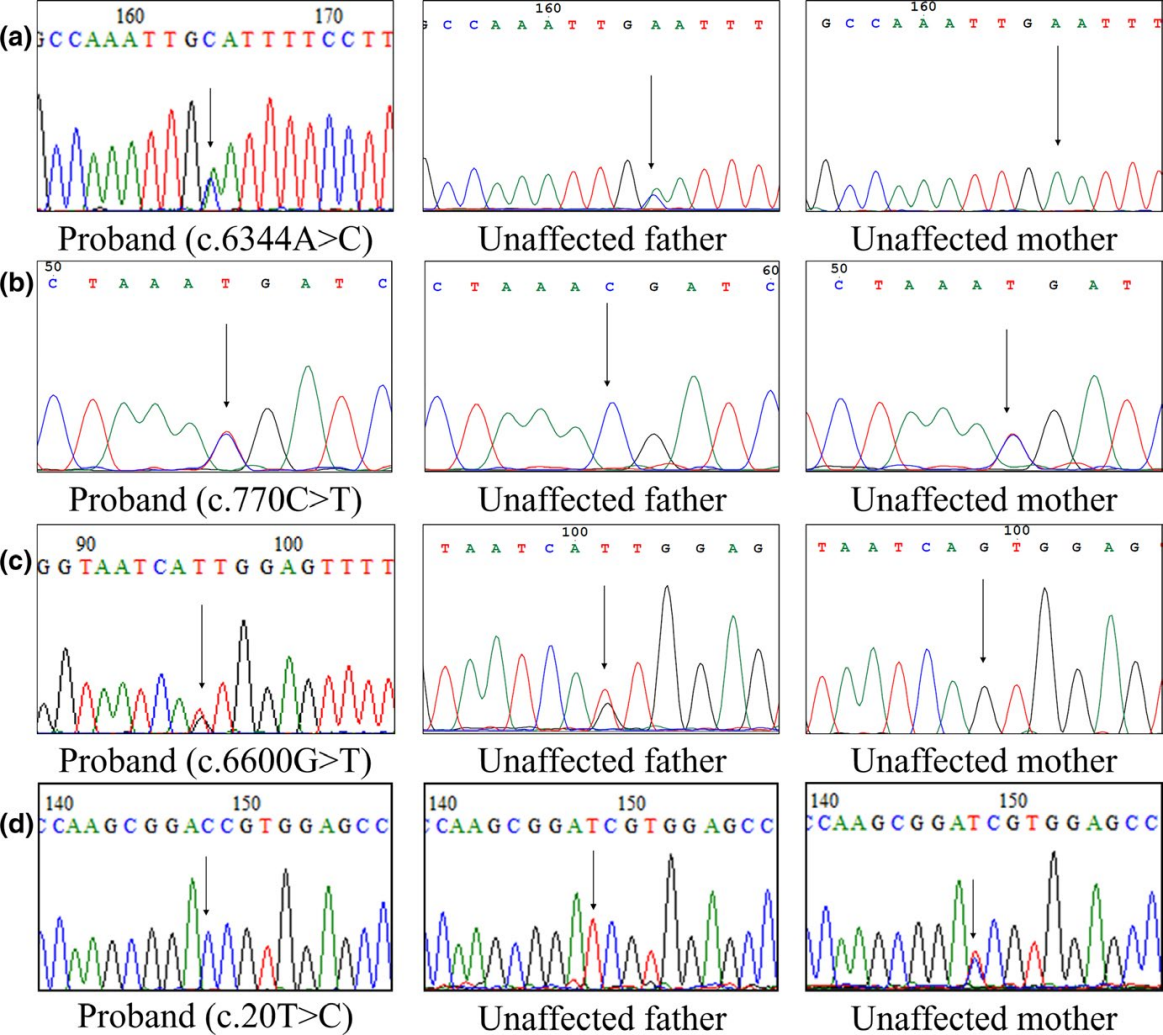
Chromatograms of the heterozygous missense variants in *JMJD1C* (a), *TCF12* (b), *BIRC6* (c), and de novo variant in *NHS* (d)

Patient 3, a 15‐year‐old male, had an ABC‐T score of 20 (Table 3). The 750K microarray showed a 1q21.1q21.2 duplication (1949.031 kbp) containing 10 OMIM genes (*HYDIN2*, *PRKAB2*, *FMO5*, *CHD1L*, *BCL9*, *ACP6*, *GJA5*, *GJA8*, *GPR89B*, and *NBPF11*). WES revealed seven rare variants in *WFS1* (c.2144G > T; p.S715I; rs772022154), *TNN* (c.1681T > C; p.Y561H; rs777370361), *JMJD1C* (c.6344T > G; p.F2115C; novel) (Figure 1a), *APP* (c.1748A > G; p.E583G; rs778495527), *SYNE1* (c.9878C > T; p.S3293F; rs770774159), *MPP6* (c.61G > A; p.D21N; rs771283348), and *MCC* (c.60_61insAGC; p.G21delinsSG; rs72442525) (Table 2). WFS1, TNN, APP, and MCC are involved in the negative regulation of Wnt and canonical Wnt signaling pathways (GO:0030178 and GO:0090090), negative regulation of signaling transduction (GO:0009968) and endoplasmic reticulum calcium ion homeostasis (GO:0032469). Moreover, a pathogenic mutation in the *EYS* gene (p.C2139Y) was observed.

Patient 4, a 6‐year‐old male, had an ABC‐T score of 27 (Table 3). The 750K microarray revealed no abnormalities. WES revealed seven rare variants in *TSC2* (c.5418T > G; p.F1806L; rs200004126), *SETBP1* (c.2842C > T; p.R948C; rs751366974), *TCF12* (c.770G > A; p.R257H; novel) (Figure 1b), *LZTS2* (c.1259G > A; p.R420Q; rs759282265), *BIRC6* (c.6600G > T; p.Q2200H; novel) (Figure 1c), *EPHA6* (c.527A > C; p.N176T), and *ASMT* (c.451G > A; p.G151S; rs192710293) (Table 2). TSC2, LZTS2 and BIRC6 are involved in the negative regulation of Wnt and canonical Wnt signaling pathways (GO:0030178 and GO:0090090) and negative regulation of signaling transduction (GO:0009968). No pathogenic mutations were detected.

Patient 5, a 10‐year‐old male, had an ABC‐T score of 36 (Table 3). The 750K microarray showed a 9q33.1 duplication (1090.359 kbp) containing three OMIM genes (*PAPPA*, *ASTN2*, and *TRIM32*). WES revealed one de novo variant in the *NHS* (c.20T > C; p.I7T) gene (Table 2 and Figure 1d). *NHS* p.I7T has not been previously reported. Additionally, a pathogenic mutation in the *FLG* gene (p.E2422X) was observed.

The mutations in our five patients were further confirmed by Sanger sequencing of DNA from both parents to determine the origins of mutation or to reveal de novo mutations.

## 4 DISCUSSION

In this study, WES was performed to identify possible ASD causal variants in five Taiwanese families; one novel de novo variant in one trio and rare variants in each trio were successfully identified. These genes are involved mainly in the negative regulation of Wnt and canonical Wnt signaling pathways, negative regulation of signaling transduction and endoplasmic reticulum calcium ion homeostasis. We detected no association of the ABC‐T score with a particular pathway. However, possible causal variants may be missed if located within a noncoding region; thus, WGS will be necessary in future studies.

Three ASD patients (ASD25, ASD26, and ASD27) were found to carry a novel missense variant of four genes (*JMJD1C*, *TCF12*, *BIRC6*, and *NHS*) (Table 2). *JMJD1C* encodes a putative histone demethylase and is involved in the epigenetic control of gene transcription. This study identified a variant of *JMJD1C*, c.6344T > G, which results in the substitution of phenylalanine by cysteine (p.F2115C). The p.F2115C mutation is in the JmjC domain, a domain family that is part of the cupin metalloenzyme superfamily. Mutations in this gene are associated with Rett syndrome and intellectual disability (Saez et al., 2016). *TCF12* encodes a member of the basic helix‐loop‐helix E‐protein family that recognizes the consensus‐binding site (E‐box) CANNTG. This study identified a variant, c.770G > A, which results in substitution of an arginine by histidine (p.R257H) in *TCF12*. *BIRC6* encodes an inhibitor of apoptosis protein with baculoviral inhibition of apoptosis protein repeat (BIR) and ubiquitin‐conjugating enzyme E2, catalytic (UBCc) domains. This study found a variant of *BIRC6*, c.6600G > T, which results in substitution of a glutamine by histidine (p.Q2200H). *NHS* encodes a protein with four conserved nuclear localization signals that function in brain development. This study identified a variant, c.20T > C, which results in substitution of isoleucine by threonine (p.I7T) in *NHS*. Mutations in this gene are associated with Nance–Horan syndrome (Shoshany et al., 2017). These variants were not found among the 277,264 alleles in the GnomAD database and were predicted to be damaging in silico by SIFT and to be likely damaging by Polyphen2.

Most of the variants identified in this study were found in autosomal genes, whereas one was identified in the X‐Y pseudoautosomal gene, *ASMT*, which has been reported to be associated with the autism phenotype and sleep disturbance (Cai et al., 2008; Wang et al., 2013). In the present study, we identified one reported missense variant, pG151S, in the Taiwanese population with ASD. Additionally, we detected no obvious dominant or recessive compound heterozygous mutations in ASD‐related genes.

By considering pathogenic mutations with ClinVar, we found variants in four of five probands (80%). The pathogenic mutations were detected in *SMPD1*, *FUT2*, *BCHE*, *MYBPC3*, *DUOX2*, *EYS*, and *FLG2* in four different patients (Table 4). *SMPD1* encodes a lysosomal acid sphingomyelinase that converts sphingomyelin to ceramide. Defects in this gene are a cause of Parkinson's disease (Mao et al., 2017). *FUT2* encodes a Golgi stack membrane protein and is highly associated with the development of inflammatory bowel disease (Wu et al., 2017). *BCHE* encodes a cholinesterase enzyme and is a member of the type‐B carboxylesterase/lipase family of proteins. Some of the genetic variants are prone to the development of prolonged apnea following administration of the muscle relaxant succinylcholine (Panhuizen, Snoeck, Levano, & Girard, 2010). *BCHE* p.T343fs has been reported in colon adenocarcinomas and esophageal carcinomas. *MYBPC3* encodes the cardiac isoform of myosin‐binding protein C. Mutations in *MYBPC3* are one cause of familial hypertrophic cardiomyopathy (Aurensanz Clemente et al., 2017). *MYBPC3* is one of the American College of Medical Genetics and Genomics genes. *DUOX2* encodes a glycoprotein and a member of the NADPH oxidase family. *DUOX2* mutations are the most powerful genetic predisposing factors for thyroid dyshormonogenesis (Chen et al., 2018). *EYS* is mutated in autosomal recessive retinitis pigmentosa (Mucciolo et al., 2018). *FLG2* encodes an intermediate filament‐associated protein that functions in aggregation and the collapse of keratin intermediate filaments in mammalian epidermis. Mutations in this gene are associated with ichthyosis vulgaris and atopic dermatitis (Hassani et al., 2018).

**Table 4:** Summary of the pathogenic mutations detected in this study^a^

Proband ID	Gene	Base Change	Amino Acid Change	OMIM	Gene function	Biological process (s)	Human disease	Reported link to other neurological disorders
ASD−23	*SMPD1*	c.557C > T	p.P186L	607608	Sphingomyelin phosphodiesterase 1	Nervous system development	Niemann‐Pick disease, type A and B	Parkinson's disease
	*FUT2*	c.604C > T	p.R202X	182100	Fucosyltransferase 2	Protein glycosylation	Vitamin B12 plasma level Crohn disease	Deficiency in vitamin B12, clinically associated with neurodegenerative disorders
	*BCHE*	c.1027dupA	p.T343fs	177400	Butyrylcholinesterase	Choline metabolic process, Neuroblast differentiation	Butyrylcholinesterase deficiency Apnea, postanesthetic, susceptibility to, due to BCHE deficiency	No
ASD−24	*MYBPC3*	c.1000G > A	p.E334K	600958	Myosin binding protein C, cardiac	Cardiac muscle contraction, Ventricular cardiac muscle tissue morphogenesis	Cardiomyopathy, hypertrophic	No
	*DUOX2*	c.1588A > T	p.K530X	606759	Dual oxidase 2	Thyroid gland development, Thyroid hormone generation	Thyroid dyshormonogenesis	No
ASD−25	*EYS*	c.6416G > A	p.C2139Y	612424	Required to maintain the integrity of photoreceptor cells.	Detection of light stimulus involved in visual perception, Skeletal muscle tissue regeneration	Retinitis pigmentosa	No
ASD−26								
ASD−27	*FLG*	c.7264G > T	c.E2422X	135940	Aggregates keratin intermediate filaments and promotes disulfide‐bond formation among the intermediate filaments during terminal differentiation of mammalian epidermis.	Cornification, Establishment of skin barrier, Keratinocyte differentiation, Multicellular organism development, Peptide cross‐linking	Ichthyosis vulgaris	No

a Pathogenic mutations, according to the ClinVar database.

In conclusion, we report on five ASD patients with rare variants and one patient with a de novo variant. However, this association study was performed with only a small number of cases; therefore, further studies with larger sample sizes are needed.

## CONFLICT OF INTEREST

The authors declare that they have no conflict of interest.
