# Supplementary material for: Chromosomal microarray and whole‐exome sequence analysis in Taiwanese patients with autism spectrum disorder
Source: Mol Genet Genomic Med. 2019 Oct 8;7(12):e996. doi: 10.1002/mgg3.996 (PMC6900387; doi:10.1002/mgg3.996)
Supplement: Supplementary file 1 [file MGG3-7-e996-s001.docx]

| **Supplementary table 1: Primer sequences used to validate candidate variants from WES.** | | |
| --- | --- | --- |
| Primer name | Sequence (5'-3') | Amplicon length |
| *SHANK3* c.3658A>G-F | TCAGCGTCCTGGACACATC | 246bp |
| *SHANK3* c.3658A>G-R | CTGGCTCTTCCTCTGAGCTG |  |
| *DNAH10* c.2800C>T-F | GGAAGGGAAATAGGCTCCAT | 334bp |
| *DNAH10* c.2800C>T-R | TTTTGGGGGATCATAACAGC |  |
| *ESR2* c.1228C>T-F | TTCAGCCTGTGACCTCTGTG | 332bp |
| *ESR2* c.1228C>T-R | GTGGGGTAGACTGGCTCTGA |  |
| *NAALADL2* c.1424G>A-F | CCATCATCACACTGCACACA | 168bp |
| *NAALADL2* c.1424G>A-R | GAGCCAATATTGCCAAAAGC |  |
| *DLGAP3* c.1759G>C-F | CCAGGGATGGTCTTGATGAT | 179bp |
| *DLGAP3* c.1759G>C-R | CAGAGCAGCACCGACTCC |  |
| *SLC1A2* c.1091G>A-F | CCCACCCTTCTCACCTTACA | 338bp |
| *SLC1A2* c.1091G>A-R | TTGCATGTCACATTGCTTGA |  |
| *CLTCL1* c.1061G>A-F | TATCACCTTTGGTGCAGACG | 304bp |
| *CLTCL1* c.1061G>A-R | GAGAATGTGGCTACCACTCCA |  |
| *WFS1* c.2144G>T-F | CTGGAAGGAGACCAACATGG | 249bp |
| *WFS1* c.2144G>T-R | AGCAGCTTAAGGCGACAGAG |  |
| *TNN* c.1681T>C-F | TTTGACTGTGTTCGTGCTGTC | 331bp |
| *TNN* c.1681T>C-R | GGTGTCAGCCTTCTTGCTCT |  |
| *JMJD1C* c.6344A>C-F | GCCTTAGCCTCCCAAAGAGT | 343bp |
| *JMJD1C* c.6344A>C-R | TCTGCTGTTTTGAACAACTTGAA |  |
| *APP* c.1748A>G-F | GGCTCAGGGGACTCTTACCT | 316bp |
| *APP* c.1748A>G-R | AGTAAATGGTGGCTGCTGCT |  |
| *SYNE1* c.9878C>T-F | TGGAGACGGGAAATTTTGAG | 337bp |
| *SYNE1* c.9878C>T-R | GCGTATGAAGCCATGACTGA |  |
| *MPP6* c.61G>A-F | CGGCTTGAAATTTAAGGGTTT | 322bp |
| *MPP6* c.61G>A-R | TGTGCTGAATGTTTTTATTTGC |  |
| *MCC* c.60_61insAGC-F | GGACTGATCTCGCTCCTGTC | 248bp |
| *MCC* c.60_61insAGC-R | GCGTACTCCTCCTCCCAAGT |  |
| *TSC2* c.5418T>G-F | CCTTCAGATCTGCGAGGAAG | 312bp |
| *TSC2* c.5418T>G-R | GCACCAAGCAGACAAAGTCA |  |
| *SETBP1* c.2842C>T-F | GATTTCTGCTCCCTGGACAA | 334bp |
| *SETBP1* c.2842C>T-R | AGAGCAACGGGTCATACTGG |  |
| *TCF12* c.770C>T-F | TTCAGGTGGTTGCTTTTGAA | 242bp |
| *TCF12* c.770C>T-R | ACTGTGACCAGCAGGCAAAC |  |
| *LZTS2* c.1259G>A-F | CCCTGCGAGAGGACTGTG | 334bp |
| *LZTS2* c.1259G>A-R | GAGGTGAGAAAGGGGAGGAC |  |
| *BIRC6* c.6600G>T-F | AACTGCTTTTCATGATTGACACAT | 319bp |
| *BIRC6* c.6600G>T-R | ATGGCACATCTGTCTCATGC |  |
| *EPHA6* c.527A>C-F | AATCATTTCTGTTTAATCACTGTGG | 308bp |
| *EPHA6* c.527A>C-R | AATTCCGTGGGACTCATCTG |  |
| *ASMT* c.451G>A-F | TGTGGGGTATAGCTCCGTTC | 232bp |
| *ASMT* c.451G>A-R | GGGGTACTTACCACCAAGGTC |  |
| *NHS* c.20T>C-F | AGGCAAGGTGAGCAGAGAAG | 360bp |
| *NHS* c.20T>C-R | GTGGCAGCCCTGAAGGTG |  |
